# µ-Raman Spectroscopic Temperature Dependence Study of Biomimetic Lipid 1,2-Diphytanoyl-sn-glycero-3-phosphocholine

**DOI:** 10.3390/biomimetics10050308

**Published:** 2025-05-11

**Authors:** Carmen Rizzuto, Antonello Nucera, Irene Barba Castagnaro, Riccardo C. Barberi, Marco Castriota

**Affiliations:** 1 Department of Physics, University of Calabria Ponte Bucci, Cubo 33B, 87036 Rende, CS, Italy; 2 CNR-Nanotec c/o Department of Physics, University of Calabria Ponte Bucci, 87036 Rende, CS, Italy

**Keywords:** Raman spectroscopy, lipid, biomimetic system, choline, biomembranes, deuterium solution

## Abstract

Raman spectroscopy is one of the best techniques for obtaining information concerning the physical–chemical interactions between a lipid and a solvent. Phospholipids in water are the main elements of cell membranes and, by means of their chemical and physical structures, their cells can interact with other biological molecules (i.e., proteins and vitamins) and express their own biological functions. Phospholipids, due to their amphiphilic structure, form biomimetic membranes which are useful for studying cellular interactions and drug delivery. Synthetic systems such as DPhPC-based liposomes replicate the key properties of biological membranes. Among the different models, phospholipid mimetic membrane models of *lamellar vesicles* have been greatly supported. In this work, a biomimetic system, a deuterium solution (50 mM) of the synthetic phospholipid 1,2-diphytanoyl-sn-glycero-3-phosphocholine (DPhDC), is studied using μ-Raman spectroscopy in a wide temperature range from −181.15 °C up to 22.15 °C, including the following temperatures: −181.15 °C, −146.15 °C, −111.15 °C, −76.15 °C, −61.15 °C, −46.15 °C, −31.15 °C, −16.15 °C, −1.15 °C, 14.15 °C, and 22.15 °C. Based on the Raman evidence, phase transitions as a function of temperature are shown and grouped into five classes, where the corresponding Raman modes describe the stretching of the (C−N) bond in the choline head group (gauche) and the asymmetric stretching of the (O−P−O) bond. The acquisition temperature of each Raman spectrum characterizes the rocking mode of the methylene of the acyl chain. These findings enhance our understanding of the role of artificial biomimetic lipids in complex phospholipid membranes and provide valuable insights for optimizing their use in biosensing applications. Although the phase stability of DPhPC is known, the collected Raman data suggest subtle molecular rearrangements, possibly due to hydration and second-order transitions, which are relevant for membrane modeling and biosensing applications.

## 1. Introduction

The main biological functions of cell membranes are carried out due to the presence of phospholipids, which act as their essential constituents. Throughout phospholipids, the cells interact with other biological molecules inside or outside the cell. The structure of phospholipids is quite interesting, as it characterizes their biological functions. In fact, phospholipids are composed of a hydrophilic head containing a water-soluble phosphate group (called choline, the head group) and two (or more) hydrophobic alkyl chains (the tails) that are not soluble in water. They can create stable barriers and/or isolate different compartments inside the cell. Of course, all biological functions of lipids also depend on the arrangement of biomolecules (or order degree or phases) in such systems (thermodynamic phase), which depends on the intermolecular interactions between them. Nevertheless, due to the complexity of these systems, before beginning to work on living cells, it is helpful to work on representative living systems that can be made through synthetic procedures, often called biomimetic systems [1]. The term “biomimetic” describes artificial systems that can imitate the behavior of living systems, serving as powerful tools for studying membrane dynamics and biological processes such as drug delivery [2]. In recent years, many bioinspired materials have been developed, with different functions, depending on the application field for which they were designed; for example, structural support, sensing, catalysis, molecular recognition, self-assembly, and self-organization, among others [3]. DNA biological materials, due to their self-assembling properties, have attracted the interest of many researchers in the fields of photonics and bio-electronics at the nanoscale [4,5]. Studies of systems made of peptides absorbed on inorganic materials have many applications in fields such as catalysis and medicine [6,7]. Biological living materials are examples of innovative materials; in fact, they react to external stimuli by changing their shape, phases, or mechanical properties [8]. Given the behaviors of these membranes (as smart materials), and as many genes encode the proteins of membranes, studies of such systems are quite relevant. For these reasons, one of the more significant goals of in vitro studies is to find suitable native mimetic lipid membranes that are stable, at least in a certain range of temperatures [9].

Among the different models, phospholipid mimetic membrane models of *lamellar vesicles* have been greatly supported [10]. *Vesicles* can be made of one layer or two layers, called *liposomes,* and are often described as multilamellar vesicles (MLV_s_). In these vesicles, phospholipid molecules are arranged in a spherical geometry and, inside these spheres, a small amount of water remains trapped inside the liposome. The water inside the liposomes allows, for example, ions to pass through the membrane of the vesicles, simulating the behaviors of the biomembrane in real cells. Such a simple cell model allows us, by studying the simplest systems, to obtain information on the processes that take place at membrane interfaces [11].

Vesicles, described as MLV_s_, could be the origin of the formation of homogeneous liposomes, called SUV_s_ (small unilamellar vesicles with a ray in the range of 20 to 50 nm) or LUV_s_ (large unilamellar vesicles with a ray in the range of 100 to 1 µm), depending on the ray of the spheres. Furthermore, controlling hydration enables us to obtain liposomes with a ray larger than 1 µm, called GUV_s_ (giant unilamellar vesicles) [11]. Lipid bilayers arranged in vitro have to replicate the key properties of biological membranes, such as the bilayer size and shape, and the capacity to accommodate functional transmembrane proteins [12]. For example, functional analyses of H^+^−ATPases are typically performed in vesicles of lower dimensions, such as SUV_s_ and LUV_s_, which allow for proton accumulation inside the liposomes [13]. The relative proton pump test has been quite useful in monitoring the roles of some proteins and their transport mechanisms [14]. However, GUV_s_ offer additional advantages with respect to SUV_s_ and LUV_s_ for these studies as, with diameters in the range between 1 and 100 µm, GUVs present sizes that are compatible with eukaryotic cells, allowing, for example, direct observation of transport activities through the use of a conventional microscope [14].

Liposomes made with DPhPC phospholipids seem to have good potential for diagnostic and therapeutic applications [15].

In this framework, the study of phospholipid membranes in vitro is shown. In particular, phospholipid bilayers are studied in deuterated water. Oftentimes, deuterated phospholipids have been adopted as spectroscopic probes for the interpretation of biomembrane structures through comparisons with the corresponding non-deuterated phospholipid bilayers based on some structural indicators of conformational and lateral orders [16,17,18,19,20,21,22].

Phospholipid bilayers in water show three phases: L_β_, P_β′_, and L_α_ (*gel*, *ripple*, and *liquid crystalline*, respectively) [10]. Starting from the lower temperature, when the phospholipid bilayer system is in the *gel* phase, the acyl chains are arranged in a rigid configuration. The L_β_ structural order remains until the temperature does not become greater than the chain-melting temperature, when the acyl chains change their order degree and the whole system becomes more disordered, leading to a *lamellar liquid crystal* phase. The *ripple* phase is an *intermediate phase* with mixed properties of the other two phases, such as the long-range periodicity of the structure in the bilayer plane [10].

**Figure 1:**
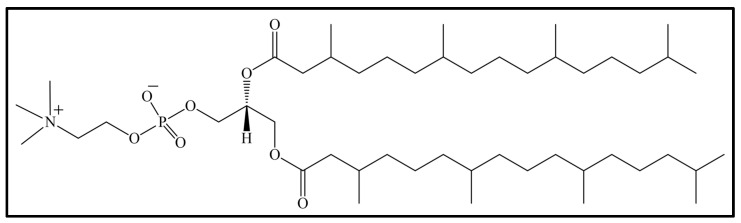
Chemical structure of 1,2-diphytanoyl-sn-glycero-3-phosphocholine (DPhPC).

The phospholipid 1,2-diphytanoyl-sn-glycero-3-phosphocholine (DPhPC) shown in Figure 1 is a synthetic lipid that, once dissolved in water, can be used to prepare bioinspired membrane systems based on phospholipids due to the conformation and orientation of the branched acyl chains [23]. In addition, the lipid shows suitable electrical properties and is also characterized by low permeability with respect to ions and water molecules. DPhPC solutions show high chemical stability due to the entirely saturated alkyl chains of the lipid, which are less reactive to the action of air or light than unsaturated chains. In addition, DPhPC solutions do not exhibit a *gel*-to-*fluid* phase transition in the wide temperature range from −120 °C to 120 °C [24].

Mimetic membranes of phospholipids have been studied using many different techniques, such as DSC [16], IR spectroscopy [25], circular dichroism (CD) [24], NMR [24,26], and others. Among the many different techniques, a quite interesting technique for studying bilayer membranes is µ-Raman spectroscopy [10]. µ-Raman spectroscopy, based on materials’ inelastic scattering of incident photons, is a non-destructive analysis technique that does not require any prior sample manipulation. It offers higher spatial resolution than conventional Raman spectroscopy, enabling the analysis of small-scale features in biomimetic systems such as liposomes or lipid bilayers. It provides detailed molecular information, allowing for the study of structural changes and interactions without invasive methods. It has been widely used in materials science to characterize several different systems such as carbon-based materials [27,28,29,30,31], polymers [32,33], metal oxide thin films [34], bi-dimensional systems [35,36], biological systems [37,38], art objects [39,40,41], and surfaces for Surface-Enhanced Raman Spectroscopy (SERS) applications [42,43,44,45]. In recent years, many studies have been performed on the characterization of biomimetic systems using SERS [46,47,48,49,50].

In this study, µ-Raman spectroscopy was employed to characterize biomimetic phospholipid DPhPC systems prepared in a 50 mM deuterium water solution across a temperature range from −181.15 °C to 22.15 °C. Within this framework, we propose the use of this sensitive spectroscopic technique to gain deeper insights into the temperature-dependent interactions between the synthetic lipid and the solvent, the conformation, the packing, and phase behavior of the lipid molecule, and the temperature-dependent phase transitions. The temperature evolution of the Raman spectra are shown and the relative attributions of the Raman bands have been carried out while taking into account different parts of the molecule: the polar head group, the stretching of the C−C backbones, the stretching of the methylene of the acyl chains, the stretching of the terminal methyl group in the acyl chains and the choline head group. Some considerations regarding the order degree of the studied mimetic system as a function of the temperature also account for the phase transitions observed.

## 2. Materials and Methods

A sample of the synthetic lipid 1,2-diphytanoyl-sn-glycero-3-phosphocholine (DPhDC) (see Figure 1) was prepared in deuterium water at a concentration of 50 mM. DPhPC was dissolved in deuterium water to reduce interferences during the Raman measurements and improve the study of the lipid/solvent system. In particular, the presence of deuterium allows us to discriminate between interactions that involve the hydrogen atoms of the phospholipid with respect to those of the water (H_2_O). In fact, in a deuterium solution, all signals assigned to hydrogen bonds are ascribed to the lipid structure, whereas in H_2_O solution, the signals of O−H from the water can overlap with those from the lipid. However, fortunately, the O–D vibrations, present in deuterated solution, fall in a down-shifted frequency region with respect to O–H vibrations, allowing clearer observation of the Raman peaks of the lipid, solvent, and their interactions.

Raman spectra were collected using a Jobin Yvon micro-Raman LABRAM equipped with a CCD (256 × 1024 pixels) detector cooled at −70 °C and an external Nd:YAG (λ = 532 nm emission, 50 mW power) laser source. A 50x Olympus objective was used to explore the surface of the samples and to collect the Raman spectra. The spectral resolution was about 1 cm^−1^. The temperature was controlled by placing the samples in a stage of the LINKAM THMS 600 cell. The Linkam stage uses liquid nitrogen to cool down the temperature of the samples and suddenly heat those to the target temperature rate. Each time the samples are at the required temperature, many Raman spectra are collected to obtain the most representative spectra of the state of the phospholipid system. The measurements were taken at a thermal range from −181.15 °C up to 22.15 °C (room temperature), using a heating rate of 1 °C/min. Raman spectra were collected at the following temperatures: −181.15 °C, −146.15 °C, −111.15 °C, −76.15 °C, −61.15 °C, −46.15 °C, −31.15 °C, −16.15 °C, −1.15 °C, 14.15 °C, and 22.15 °C.

The temperatures listed are nominal values. Such temperature values are given with this precision in order to guarantee the reproducibility of the experimental conditions and, consequently, the experimental results. In addition, such precision allows for accurate comparisons of Raman spectra collected at closely spaced temperatures.

Moreover, Raman spectra were collected between 150 and 3500 cm^−1^. The μ-Raman investigations were carried out on the following biomimetic lipid samples: 1,2-diphytanoyl-sn-glycero-3-phosphocholine (DPhPC), as the powder used as the starting reference, and the deuterium solution in a concentration of 50 mM of DPhPC.

## 3. Results and Discussion

**Figure 2:**
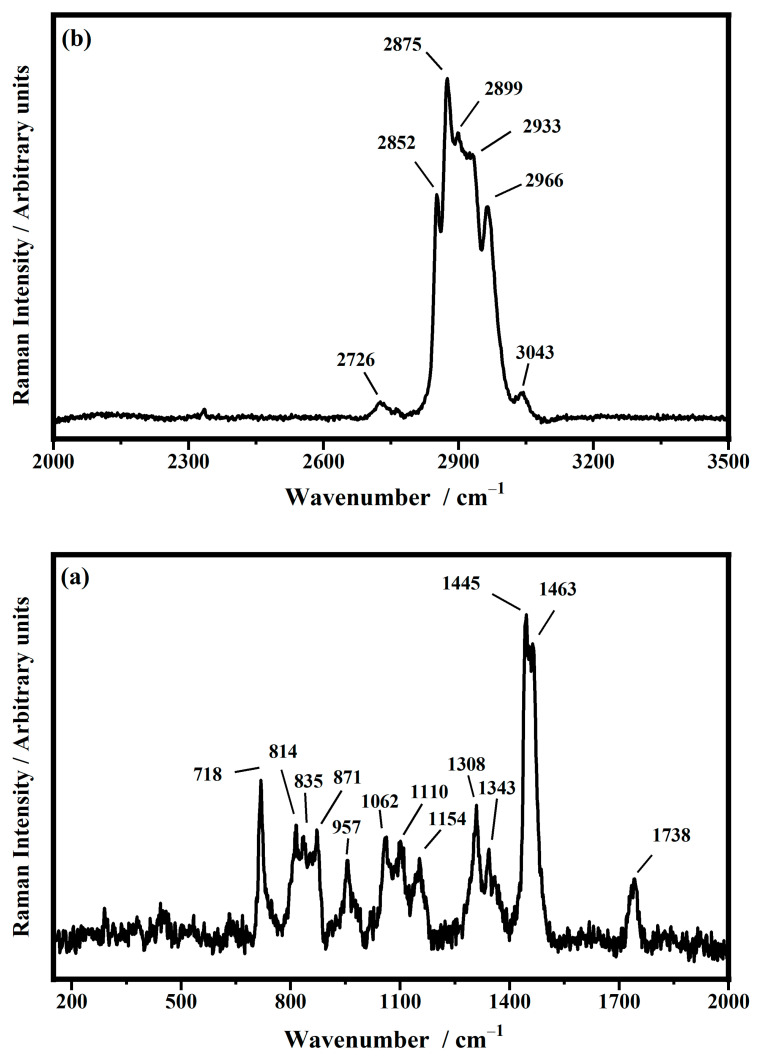
Representative Raman spectra collected on the 1,2-diphytanoyl-sn-glycero-3-phosphocholine (DPhPC) powder at room temperature in the ranges between 150 cm^−1^ and 2000 cm^−1^ (**a**) and between 2000 cm^−1^ and 3500 cm^−1^ (**b**).

The structure of the synthetic lipid 1,2-diphytanoyl-sn-glycero-3-phosphocholine (DPhDC) and the representative spectrum collected on the powder of the phospholipid (DPhDC) are shown in Figure 1 and Figure 2, respectively.

The Raman spectrum of DPhPC in powder is used as a reference for the spectra of DPhPC in solutions in order to show the representative Raman features of the phospholipid, their attributions to the relative vibrational normal modes, and how these modes are affected by the surrounding environment. This protocol clarifies how the structure and interactions of the lipid molecules change when moving from powder to a hydrated environment, similar to real biological conditions.

The Raman spectrum (Figure 2a) relative to the fingerprint region, ranging between 150 and 2000 cm^−1,^ shows some characteristic bands of DPhPC lipid powder. The bands that fall at 718 cm^−1^ and 871 cm^−1^ are assigned to the vibrational stretching of the C−N bond of the choline head group (O−C−C−N^+^) in the *gauche* conformation [10,16,51,52,53,54,55,56]. The bands at about 835 cm^−1^ and 853 cm^−1^ are assigned to the asymmetric stretching of the O−P−O bond in the glycerophosphorylcholine head group [10,17,45,46,47,48,49,50]. The vibrational peak at 957 cm^−1^ is ascribed to the rocking mode of the methylene groups. The lipid hydrocarbon acyl chains are detected for the modes associated with the skeletal vibrations of the C−C bonds at about 1062 cm^−1^, 1097 cm^−1^, and 1110 cm^−1^, as broad bands. In particular, the asymmetric stretching of the C−C bonds in the *trans* conformation is found at 1062 cm^−1^. The Raman mode at 1097 cm^−1^ is assigned to the stretching of the C−C bonds in the *gauche* conformation and includes the phosphate stretching mode of the head group of the lipid as well. The Raman mode at 1110 cm^−1^ is assigned to the *trans* C−C bonds, while the stretching mode of the C−C bonds is found at 1154 cm^−1^. The Raman features at about 1308 cm^−1^ and 1463 cm^−1^ are assigned to the CH_2_ scissor mode, while the band at 1445 cm^−1^ is assigned to the twisting mode of the methylene groups. The band at 1308 cm^−1^ can be due to the twisting vibrational mode of the CH_2_ groups, too. The stretching mode of C=O ester groups is found at 1738 cm^−1^.

Figure 2b shows the Raman spectrum in the range between 2000 and 3500 cm^−1^.

The peak at 2726 cm^−1^ is due to the C−H stretching vibration of CH_2_. The symmetric stretching of the methylene group is assigned to the band at 2852 cm^−1^, while that of the CH_2_ groups falls at 2899 cm^−1^. The Raman feature at 2875 cm^−1^ is attributed to asymmetric methylene CH_2_ stretching. The vibrational band at 2933 cm^−1^ is due to the stretching mode of the CH_3_ terminal methyl groups. The Raman features that fall at 2966 cm^−1^ and 3043 cm^−1^ are assigned to the methyl asymmetric stretching modes associated with the choline moiety, N−(CH_3_)_3_ [10,16,51,52,53,54,55,56]. All vibrational modes of the DPhPC powder are summarized in Table 1.

Exploring the synthetic lipid DPhDC in a deuterium solution using Raman spectroscopy provides several biomimetic insights, particularly when considering its interaction with membranes and the solvent, as well as at the interface. In this framework, the investigation of vibrational modes offers detailed insight into the phase behavior of the investigated DPhDC, helping us understand how this artificial model, which simulates the natural phospholipids in biological membranes, behaves.

**Figure 3:**
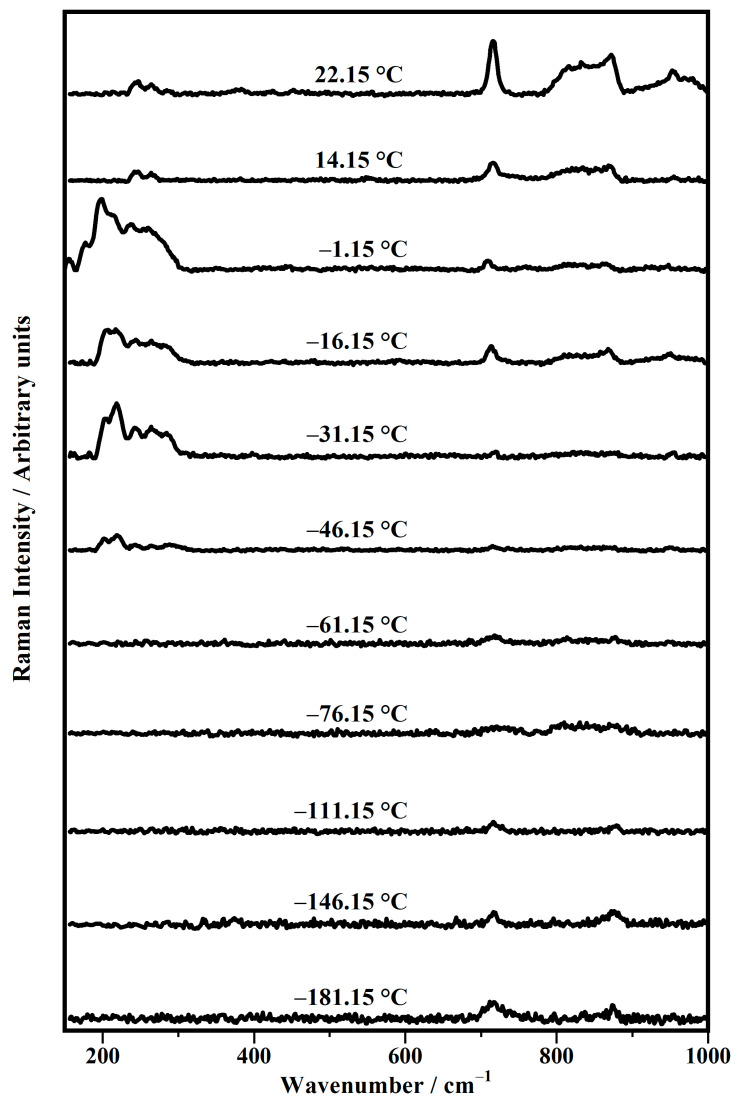
Temperature evolution in the range between −181.15 °C and 22.15 °C of representative Raman spectra, collected on the 1,2-diphytanoyl-sn-glycero-3-phosphocholine (DPhPC) in deuterium solution (50 mM) in the range between 150 cm^−1^ and 1000 cm^−1^.

Figure 3 shows the representative Raman spectra, between 150 cm^−1^ and 1000 cm^−1^, collected on the DPhPC prepared in a deuterium solution (50 mM), as a function of the temperature in a range between −181.15 °C and 22.15 °C (room temperature).

The spectra shown in Figure 3, based on their Raman features, can be grouped into five classes: spectra collected at (a) −181.15 °C and −146.15 °C; (b) −111.15 °C, −76.15 °C, and −61.15 °C; (c) −46.15 °C, −31.15 °C, and −16.15 °C; (d) −1.15 °C; and (e) 14.15 °C and 22.15 °C.

Regarding the spectra of class (a) (at −181.15 °C and −146.15 °C), two Raman modes can be detected at 717 cm^−1^, assigned to the *gauche* conformation of the choline head group, and the mode at 872 cm^−1^, assigned to the stretching mode of the C−N bond of choline ν(C−N). In class (b) (at −111.15 °C, −76.15 °C, −61.15 °C), such Raman features become negligible, while in class (c) (at −46.15 °C, −31.15 °C, −16.15 °C), some Raman features below 300 cm^−1^ are increased, and are assigned to lattice vibrations and longitudinal acoustic vibrations of extended chains, probably due to phosphate deformations [56]. Again, the two Raman modes are at 717 cm^−1^ and at 872 cm^−1^, assigned as mentioned above, together with the band at about 830 cm^−1^, ascribed to the asymmetric stretching of the O−P−O bonds. A small peak at 953 cm^−1^ can be observed on these spectra and attributed to the rocking mode (ρ CH_2_) of the methylene of the acyl chains. The spectrum of class (d) (at −1.15 °C) shows Raman features similar to those of spectra of class (c), with the only difference in the features below 300 cm^−1^, where a downshift in the main bands can be seen.

The spectra grouped in the last class (e) (at 14.15 °C, 22.15 °C) show small bands at 243 cm^−1^ and 264 cm^−1^, with different profiles with respect to those seen in the spectra in the previous classes. In addition, the band at 717 cm^−1^ becomes stronger than those shown above, together with those Raman bands that fall at 830 cm^−1^, 872 cm^−1^, and 953 cm^−1^.

The corresponding Raman modes are summarized in Table 2 as a function of the acquisition temperature of each Raman spectrum.

**Figure 4:**
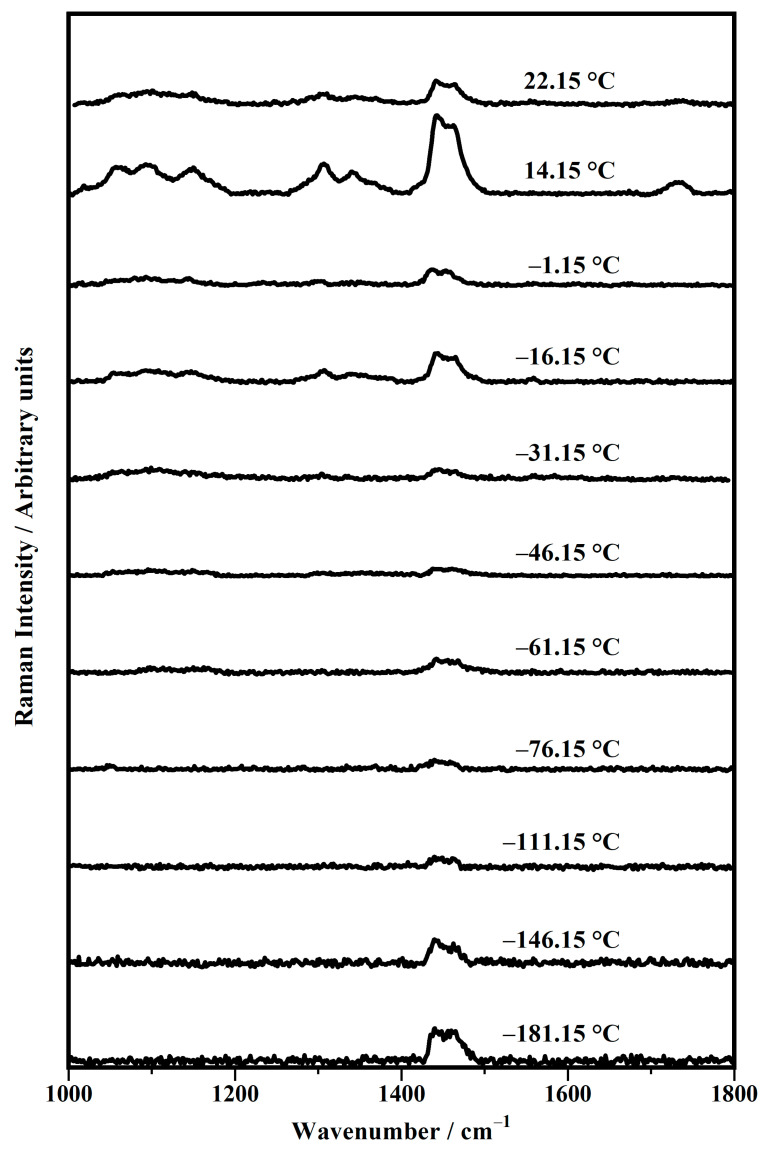
Temperature evolution in the range between −181.15 °C and 22.15 °C of representative Raman spectra, collected on the 1,2-diphytanoyl-sn-glycero-3-phosphocholine (DPhPC) in deuterium solution (50 mM) in the range between 1000 cm^−1^ and 1800 cm^−1^.

Figure 4 shows the representative Raman spectra, between 1000 cm^−1^ and 1800 cm^−1^, collected on the DPhPC prepared in a deuterium solution (50 mM) as a function of the temperature in a range between −181.15 °C and 22.15 °C (room temperature).

Similar to what is observed above, with regard to Figure 3, the spectra shown in Figure 4 are also grouped into the five classes: spectra collected at (a) −181.15 °C and −146.15 °C; (b) −111.15 °C, −76.15 °C, and −61.15 °C; (c) −46.15 °C, −31.15 °C, and −16.15 °C; (d) −1.15 °C; and (e) 14.15 °C and 22.15 °C.

The Raman spectra of class (a) (at −181.15 °C and −146.15 °C) show two Raman peaks at about 1442 cm^−1^ and 1464 cm^−1^, assigned to the twisting (τ CH_2_) and scissoring (σ CH_2_) modes of the methylene group of the acyl chains, respectively. Such bands decrease when passing in the spectra of class (b) (at −111.15 °C, −76.15 °C, −61.15 °C) and increase again in the spectra of class (c) (−46.15 °C, −31.15 °C, −16.15 °C). In class (d) (at −1.15 °C), such modes decrease again and then become quite intense in the spectrum of class (e) (at 14.15 °C, 22.15 °C) (Table 3).

The behavior of the twisting (τ CH_2_) and scissoring (σ CH_2_) modes describes the presence of other components in the Raman features attributable to the C−C backbone vibrations. In the high-temperature spectra of class (c), the C−C stretching in the *gauche* conformation, the stretching of the C−C vibrations in the *trans* conformation, and the scissoring of the C−C bonds of the acyl chains become detectable. In particular, the antisymmetric C−C stretching mode (ν_as_ C−C, *trans*) of the acyl chains becomes detectable around 1060 cm^−1^, the *gauche* conformation of the C−C bonds (ν_s_ C−C) in the acyl chains is confirmed by the presence of the peaks at about 1095 cm^−1^, and the vibrational stretching of the C−C bonds (ν C−C) in the *trans* conformation falls at 1110 cm^−1^ (Figure 4). Here, a small Raman band at about 1147 cm^−1^ is assigned to the out−of−phase C−C vibrations (ν_ofp_ C−C) (Table 4). In addition, in the spectra of class (c) at higher temperatures, some small bands at 1306 cm^−1^ and 1339 cm^−1^ can always be observed. This can be due to the overlapping of two kinds of vibrational modes, the scissoring (σ CH_2_) or the twisting modes of the methylene group (τ CH_2_) and the wagging modes of the CH_2_, (ω CH_2_) (Figure 4) (Table 5).

All modes described above—such as ν_as_ (C−C) at about 1060 cm^−1^, ν_s_ (C−C) at about 1095 cm^−1^, ν (C−C) at 1110 cm^−1^, ν_ofp_ (C−C) at 1147 cm^−1^, and σ (CH_2_) and τ (CH_2_) at about 1306 cm^−1^ and 1339 cm^−1^, respectively—decrease their intensities in the spectrum of class (d) and then increase considerably in the spectra of class (e), where the band assigned to the vibrational stretching of the C=O ester group arises with the modes at about 1732 cm^−1^.

**Figure 5:**
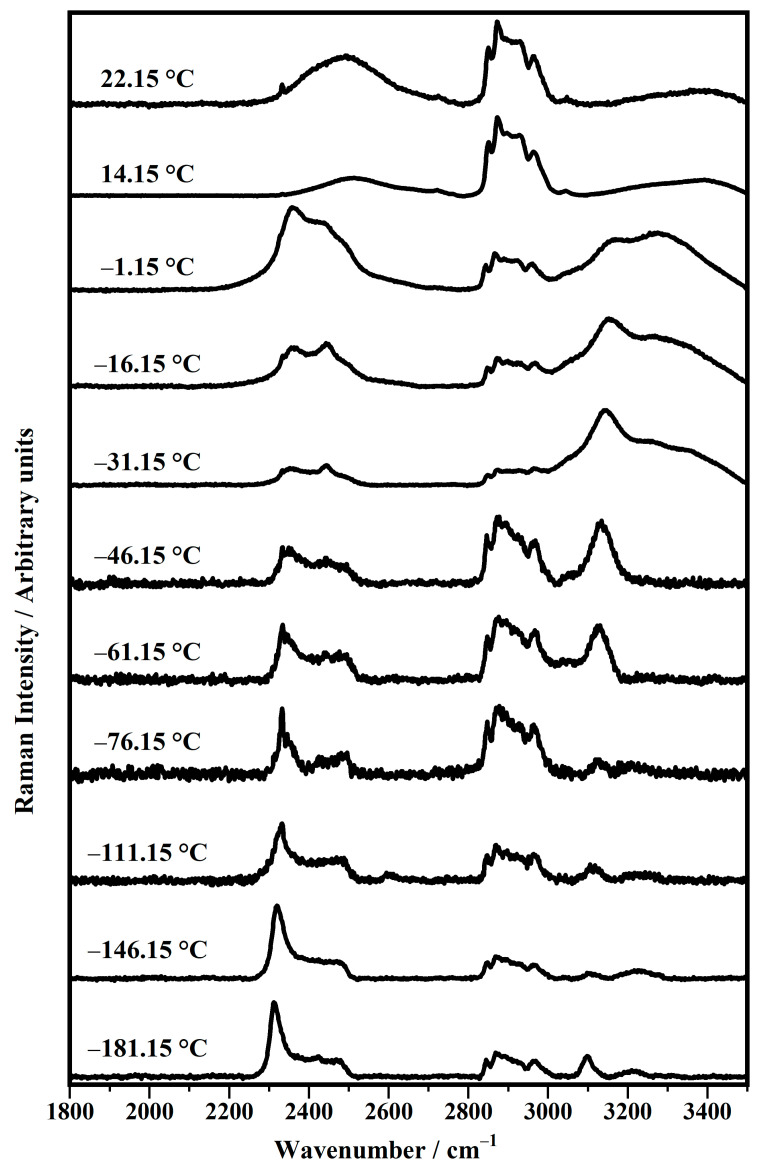
Temperature evolution in the range between −181.15 °C and 22.15 °C of representative Raman spectra, collected on the 1,2-diphytanoyl-sn-glycero-3-phosphocholine (DPhPC) in deuterium solution (50 mM) in the range between 1800 cm^−1^ and 3500 cm^−1^.

Figure 5 shows the representative Raman spectra, between 1800 cm^−1^ and 3500 cm^−1^, collected on the DPhPC prepared in a deuterium solution (50 mM) as a function of the temperature in a range between −181.15 °C and 22.15 °C (room temperature).

The classification above is confirmed by the trends in the Raman features as a function of the temperatures, as shown in Figure 5. For this reason, similarly to what is observed above, the spectra shown in Figure 5 are also grouped into the five classes: spectra collected at (a) −181.15 °C and −146.15 °C; (b) −111.15 °C, −76.15 °C, and −61.15 °C; (c) −46.15 °C, −31.15 °C, and −16.15 °C; (d) −1.15 °C; and (e) 14.15 °C and 22.15 °C.

In the Raman spectra of class (a) (at −181.15 °C and −146.15 °C), it is possible to observe a broad band between 2300 and 2600 cm^−1^ that contains the main feature related to the stretching vibration of the O−D (oxygen−deuterium) modes [57,58,59]. Here, three main Raman bands can be seen at about 2312 cm^−1^, 2420 cm^−1^, and 2480 cm^−1^ [59]. It has been hypothesized that the mode at 2312 cm^−1^ could be due to a downshift in the mode termed “*ice-like mode*” and observed in the Raman spectrum of ice [57]. The peak around 2500 cm^−1^ is ascribed to the symmetric and asymmetric deuterium−oxygen (OD) of the water, especially in the disordered phase, that is, at higher temperatures, of the liquid−water mode [58].

The spectra of class (a) also show other bands at 2847 cm^−1^, 2868 cm^−1^, 2961 cm^−1^, 3098 cm^−1^, and 3210 cm^−1^ (Figure 5). In particular, the band at about 2847 cm^−1^ is ascribed to the symmetric stretching (ν_s_) of methylene groups, and the other two bands at about 2868 cm^−1^ are attributed to the asymmetric stretching (ν_as_) of the CH_2_ groups. The band at 2961 cm^−1^ is associated with the asymmetric stretching (ν_as_) of the CH_3_ groups. The other modes at 3098 cm^−1^ and 3210 cm^−1^ are due to the asymmetric stretching (ν_sa_) modes of the methyl terminal of the choline head group and of O−H and N−H bonds, respectively [10,16,50,51,52,53,54,55].

In Figure 5, it can be seen that the spectra of class (b) (at −111.15 °C, −76.15 °C, −61.15 °C) show a similar profile to the previous ones, with the main bands that fall at upper frequencies: at 2332 cm^−1^ and 3134 cm^−1^. The other bands do not change their Raman shift, and the band at 3210 cm^−1^ is not present in the spectra of class (b). It is interesting to note how noise seems to be larger in these spectra than in the others. The other spectra of class (c) (at −46.15 °C, −31.15 °C, −16.15 °C) show that the Raman feature at 2332 cm^−1^ becomes smaller than the other bands. Even the bands between 2800 cm^−1^ and 3000 cm^−1^ decreased their intensities compared to the other bands. The bands associated with the asymmetric stretching (ν_as_) modes of the methyl terminal of the choline head group (around 3100 cm^−1^) in the spectra of class (c) continue their upshift at 3156 cm^−1^.

The spectrum of class (d) (at −1.15 °C) shows bands at 2357 cm^−1^, 2436 cm^−1^, 2480 cm^−1^, 3150 cm^−1^, and 3273 cm^−1^. These are quite wide, with increased intensities compared to those in the region between 2800 cm^−1^ and 3000 cm^−1^. The spectra of the last class (e) (at 14.15 °C, 22.15 °C) are still different from the others and show a broad band at about 2490 cm^−1^ and the other peaks in the region between 2800 cm^−1^ and 3000 cm^−1^, due to C−H stretching. All the other bands outside of the region 2800 cm^−1^ and 3000 cm^−1^ become negligible. The main Raman features are summarized in Table 6.

A significant study stated that 1,2-diphytanoyl-sn-glycero-3-phosphocholine (DPhPC) did not show any gel–liquid crystalline transition phases in the temperature range from −120 °C to +120 °C [60,61].

Nevertheless, based on the Raman features of the spectra shown above (Figure 3, Figure 4 and Figure 5), the Raman study on the function of temperature, shown in this work, suggests that the Raman spectra can be grouped into five classes, using the function of temperature at which the spectra were collected. For this reason, five classes were defined:

Class (a) contains the spectra collected at −181.15 °C and −146.15 °C;

Class (b) contains the spectra collected at −111.15 °C, −76.15 °C, and −61.15 °C;

Class (c) contains the spectra collected at −46.15 °C, −31.15 °C, and −16.15 °C;

Class (d) contains the spectrum collected at −1.15 °C;

Class (e) contains the spectra collected at 14.15 °C and 22.15 °C.

Such classes represent five “structural orders” (phases) of the studied systems, that is, how the biomolecules and the molecules of the solvents are arranged at the indicated temperatures.

The changes in the Raman spectra observed indicate that the DPhPC solution changes their internal interactions. This can be due to a change in phase or at gradual structural relaxations or local reorganizations within the same phase. Nevertheless, the structural order results are modified, and we think it is possible to use the term “phases” to indicate the same system with different structural orders.

Class (a) is the most probable to represent the *gel* phase (or the phase closest to the maximum order possible for these systems) unless the temperature does not reach −146.15 °C. The peaks are sharp, and the bands result in higher intensities. It is well known that systems with a high grade of positional order and orientation order (close to solid) show great Raman cross-sections. This explains why the Raman bands are sharper in the spectra of class (a) than in the other spectra collected at higher temperatures [62,63]. This gel phase is called FC.

Class (b) represents a new mesophase (termed FC_β_), where the positional order at a long ray starts to decrease, becoming slightly more fluid, whereas the orientation order is still present. The lipids are still in a “*gel-like*” phase in this class. In all frequency ranges (Figure 3, Figure 4 and Figure 5), it is possible to see how the noise of the spectra increases, indicating that a phase with a small Raman cross-section is present. Of course, such phase transitions can be due to different molecular packings affected by the hydration of the DPhPC molecules, resulting in arrangements with different order degrees [26]. When the samples pass from the highest temperature of class (b) (−61.15 °C) to the lowest of class (c) (−46.15 °C), there is a transition phase between the “*gel-like*” phase to an *intermediate lamellar phase* (termed FC_γ_) detectable by the presence of new bands assigned to some parts of the chains of the biomolecules, which results in increased freedom of motion [64]. Such behavior is confirmed for all spectra of class (c).

The spectrum of class (d) suggests that at this temperature, just below zero Celsius, the water molecules affect the resulting phase, which shows particular Raman features quite different from those collected at lower and higher temperatures. It represents another *intermediate lamellar phase* (termed FC_δ_), with increased mobility of molecules, as can be deduced by the presence of the broad bands from the previous phase FC_γ_.

The last class (e) shows spectra that can be assigned to the lamellar liquid crystal phase seen elsewhere [64].

Of course, the temperatures of each spectrum indicated in this work do not represent transition temperatures but temperatures at which the samples show the described phases. This means that the transition temperatures are within the ranges between two subsequent classes:

**Class (a)** T = −146.15 °C **→ Class (b)** T = −111.15 °C; T = −61.15 °C **→ Class (c)** T = −46.15 °C, T = −16.15 °C **→ Class (d)** T = −1.15 °C **→ Class (e)** T = +14.15 °C

The results of the present work seem to contradict what was stated above concerning the absence of any gel-to-fluid phase transition in the wide temperature range from −120 °C to 120 °C [24,60]. Nevertheless, other authors suggest that some “phases transitions” depend on the hydration degree of the phospholipid, which may be undetectable with other techniques, supporting our conclusions [65]. As stated above, the liposomes change in diameter as a function of the hydration, passing from the SUV_s_, to the LUV_s_, and up to the GUV_s_ that enclose most of the water [11,12].

In addition, the first phase (FC) shown in this work is below −120 °C (class (a)), which does not contradict the results of the literature [60]. The other transitions that occur between the different classes indicated above may not have been detected with other techniques since they are phase transitions of the second order, which do not occur with the “traditional” high-latency enthalpy typical of transitions of the first order. Understanding these transitions helps in designing membranes with tunable fluidity and permeability, which is vital for biosensing, synthetic cell models, and responsive biomaterials.

Lastly, the detailed phase behavior of DPhPC characterized through Raman spectroscopy in a D_2_O environment, as presented in this study, provides a foundational framework for further investigations using complementary structural techniques. Notably, the use of D_2_O is highly advantageous for diffraction-based methods such as Neutron Reflectometry (NR), which rely on contrast variation enabled by hydrogen–deuterium substitution. As shown by Villanueva et al. [66], NR can reveal critical details about the out-of-plane structure and hydration dynamics of lipid bilayers, particularly in the presence of nanomaterials. Moreover, understanding thermally induced structural transitions is essential when integrating spectroscopic and diffraction data, as highlighted in studies of other lipid systems by Kučerka et al. [67] and Disalvo et al. [68]. These insights collectively underscore the relevance of DPhPC-based biomimetic membranes as model systems for probing biophysical membrane properties across multiple experimental modalities.

## 4. Conclusions

In this work, a biomimetic system, comprising a deuterium solution (50 mM) of the synthetic phospholipid 1,2-diphytanoyl-sn-glycero-3-phosphocholine (DPhDC), was studied using µ-Raman spectroscopy in a wide temperature range from −181.15 °C up to 22.15 °C at the following temperatures: −181.15 °C, −146.15 °C, −111.15 °C, −76.15 °C, −61.15 °C, −46.15 °C, − 31.15 °C, −16.15 °C, −1.15 °C, 14.15 °C, and 22.15 °C. Raman spectra were collected in the range between 150 cm^−1^ and 3500 cm^−1^.

The attributions of the characteristic vibrational modes of the DPhPC powder were shown and used as references for the spectra collected on the deuterated lipid solution.

Spectra were collected at various temperatures, and five distinct classes were identified based on the Raman features.

Class (a) includes spectra from −181.15 °C and −146.15 °C, representing a gel phase with sharp Raman peaks, indicating a high order in the system. Class (b) covers temperatures from −111.15 °C to −61.15 °C and corresponds to a mesophase with reduced positional order but intact orientation. In this phase, the lipids are still in a “*gel-like*” phase. Class (c) is observed at temperatures between −46.15 °C and −16.15 °C, where the system transitions from gel-like to an intermediate lamellar phase, with new bands indicating changes in the lipid chains. Class (d), at −1.15 °C, reflects a phase with higher molecular mobility and increased interactions with water molecules, as indicated by broad Raman bands. Finally, class (e) at 14.15 °C and 22.15 °C shows spectra typical of the lamellar liquid crystal phase.

These classes represent different structural phases, with transitions occurring between them. The temperature ranges do not correspond to exact phase transition temperatures, but to the observed phases. This study suggests that DPhPC does not exhibit a simple gel-to-fluid phase transition, as expected from the previous literature, and highlights the potential detection challenges of intermediate phase transitions in hydrated biomimetic systems, which may not exhibit the traditional sharp transitions seen in first-order phase changes.

The development of synthetic lipids can be engineered to mimic specific structural phases found in biological membranes, which is crucial for designing membrane models or drug delivery systems.

Future detailed studies are planned that will match the Raman data that will be collected with a reduced temperature step, together with other techniques; for example, TGA, Raman polarization, CD, and others. These methods could be further optimized for application in biosensors, where membrane alterations induced by analyte binding lead to detectable Raman shifts.

## Figures and Tables

**Table 1:** Raman modes of DPhPC powder collected in the range between 150 cm^−1^ and 3500 cm^−1^.

Band Position/cm^−1^	Raman Modes
718	Stretching C−N (choline *gauche* conformation)
835	Asymmetric stretching O−P−O
853	Asymmetric stretching O−P−O
871	Stretching C−N (choline *gauche* conformation)
957	Rocking CH_2_
1062	Skeletal vibration C−C (asymmetric C−C) of the acyl chains
1097	Stretching C−C (*gauche* C−C conformation)/Symmetric stretching PO_2_-
1110	Skeletal C−C vibration (*trans*-C−C conformation)
1154	Stretching C−C
1308	Scissoring CH_2_/Twisting CH_2_
1445	Twisting CH_2_
1463	Scissoring CH_2_
1738	Stretching C=O (ester group)
2726	Stretching C−H (CH_2_ group)
2852	Symmetric stretching CH_2_
2875	Asymmetric stretching CH_2_
2899	Asymmetric stretching CH_2_
2933	Stretching CH_3_ terminal
2966	Asymmetric stretching CH_3_ (choline head group)
3043	Asymmetric stretching CH_3_ (choline head group)

**Table 2:** Where frequencies fall: the stretching of C−N bond, ν (C−N), in the choline head group, the asymmetric stretching of O−P−O bonds, ν_as_ (O−P−O), and the rocking of CH_2_ (ρ CH_2_) in a deuterium solution of DPhPC (50 mM) collected at different temperatures in the range between 150 cm^−1^ and 1000 cm^−1^.

Temperature/°C	ν (C−N) Choline (*Gauche*)	ν_as_ (O−P−O)	ν (C−N) Choline (*Gauche*)	ρ CH_2_
−181.15	718	/	874	/
−146.15	716	/	875	/
−111.15	716	/	874	/
−76.15	721	831	879	/
−61.15	718	831	877	/
−46.15	715	831	876	949
−31.15	719	828	875	954
−16.15	717	830	872	955
−1.15	709	831	865	947
14.15	717	834	869	955
22.15	717	834	874	950

**Table 3:** Where frequencies fall: the scissoring (σ CH_2_) and the twisting (τ CH_2_) vibrational modes in a deuterium solution of DPhPC (50 mM) collected at different temperatures in the range between 1000 cm^−1^ and 1800 cm^−1^.

Temperature/°C	σ (CH_2_)	τ (CH_2_)
−181.15	1440	1464
−146.15	1440	1462
−111.15	1440	1461
−76.15	1440	1458
−61.15	1443	1469
−46.15	1444	1460
−31.15	1440	1465
−16.15	1444	1465
−1.15	1437	1455
14.15	1440	1465
22.15	1443	1463

**Table 4:** Where frequencies fall: the asymmetric stretching of C−C (ν_as_ C−C, *trans*), symmetric stretching of C−C (ν_s_ C−C, *gauche*), stretching of C−C (ν C−C, *trans*), and out−of−plane C−C stretching (ν_ofp_ C−C) in a deuterium solution of DPhPC (50 mM) collected at different temperatures in the range between 1000 cm^−1^ and 1800 cm^−1^.

Temperature/°C	ν (C−C) (*Trans*)	ν_s_ (C−C) (*Gauche*)	ν (C−C) (*Trans*)	ν_ofp_ (C−C)
−46.15	1060	1095	1110	1148
−31.15	1063	1092	1110	1145
−16.15	1062	1091	1110	1147
−1.15	1063	1092	1110	1145
14.15	1064	1094	1110	1148
22.15	1060	1093	/	1151

**Table 5:** Where frequencies fall: the scissoring (σ) and wagging (ω) vibrational modes of the CH_2_ groups in a deuterium solution of DPhPC (50 mM) collected at different temperatures in the range between 1000 cm^−1^ and 1800 cm^−1^.

Temperature/°C	σ (CH_2_)	ω (CH_2_)	ν (C=O)
−46.15	1307	1343	/
−31.15	1304	1336	/
−16.15	1304	1338	/
−1.15	1306	1339	/
14.15	1310	1345	1732
22.15	1307	1339	1732

**Table 6:** Where frequencies fall: the O−D modes in the range between 2300 cm^−1^ and 2600 cm^−1^, symmetric stretching ν_s_ (CH_2_) and asymmetric stretching ν_as_ (CH_2_) of CH_2_ groups, symmetric stretching ν_s_ (CH_3_) of the CH_3_ terminal groups in the acyl chains, asymmetric stretching ν_as_ [N-(CH_3_)_3_] of the methyl terminal groups of the chains in the choline head group, asymmetric stretching ν_as_ (CH_3_) of the terminal CH_3_ in the choline head group in a deuterated solution of DPhPC (50 mM) collected in a deuterium solution of DPhPC (50 mM) collected at different temperatures in the range between 1800 cm^−1^ and 3500 cm^−1^.

Temperature/°C	ν_s_ (O−D)	ν_s_ (O−D)	ν_s_ (O−D)	ν_s_ (CH_2_)	ν_as_ (CH_2_)	ν_as_ (CH_3_)	ν_as_ [N-(CH_3_)_3_]	ν_s_ (O−H &N−H)
−181.15	2312	2420	2476	2844	2868	2969	3093	3210
−146.15	2320	2419	2482	2847	2866	2968	3098	/
−111.15	2332	2424	2478	2847	2869	2964	3105	/
−76.15	2333	2425	2496	2847	2877	2964	3118	/
−61.15	2334	2438	2490	2847	2876	2967	3124	/
−46.15	2333	2444	2495	2846	2872	2968	3134	/
−31.15	2353	2444	2493	2850	2875	2965	3143	3257
−16.15	2356	2443	2495	2847	2877	2968	3153	3268
−1.15	2357	2438	2494	2844	2866	2961	3165	3272
14.15	/	/	2502	2848	2875	2967	/	/
22.15	/	/	2498	2851	2873	/	/	/

## Data Availability

The data presented in this study are available upon request to the corresponding author.

## Abbreviations

The following abbreviations are used in this manuscript:

DPhPC	1,2-diphytanoyl-sn-glycero-3-phosphocholine.
